# Characteristics of adults with influenza A virus pneumonia and co-infections identified by mNGS in Jilin, China during 2024–2025

**DOI:** 10.3389/fcimb.2025.1662422

**Published:** 2025-09-30

**Authors:** Wei Li, Xin Di, Xuejiao Lv, Lin Zhang, Jinyan Yu

**Affiliations:** Department of Respiratory and Critical Care Medicine, The Second Hospital of Jilin University, Changchun, Jilin, China

**Keywords:** influenza A virus, pneumonia, hospitalized patients, co-infections, fungal infections, disease severity

## Abstract

**Introduction:**

Influenza A virus (IAV) was included in the World Health Organization priority pathogen list for 2024 owing to its pandemic potential. We aimed to investigate the characteristics of IAV pneumonia and co-infection identified using metagenomic next-generation sequencing (mNGS) in hospitalized patients in Jilin, China, during 2024–2025.

**Methods:**

This retrospective study included patients hospitalized for IAV pneumonia. All patients underwent mNGS testing using sputum or bronchoalveolar lavage fluid. Patients were categorized into mild-to-moderate (MM) and severe-to-critical (SC) groups, depending on their disease severity. We analyzed demographic data, clinical manifestations, laboratory findings, and imaging results, and compared the two groups.

**Results:**

Of the 73 patients included, 45 were in the MM group and 28 were in the SC group. Compared with nucleic acid tests of throat swabs, mNGS has higher sensitivity for detecting IAV (60% vs 100%). H1N1 and H3N2 were the predominant IAV subtypes. Underlying conditions, especially asthma and chronic obstructive pulmonary disease, were associated with an increased risk of severe illness. The D-dimer levels were higher, and lymphocyte counts were lower in patients in the SC group than in those in the MM group. Of the 73 patients, 63 (86.3%) had secondary infections, with bacterial infections being more prevalent (mild/moderate: 26 [58%] and severe/critical: 24 [86%]) than fungal infections (23 [51%] and 23 [82%], respectively).

**Conclusions:**

mNGS is a sensitive method for detecting IAV co-infections, effectively identifying co-infection with pathogenic bacterial strains. Hospitalized patients with IAV pneumonia, especially those with H3N2 infection and chronic airway disease, showed a high prevalence of severe and critical illness [total: 8 [11%], severe/critical: 7 [25%]). Fungal infections were frequent regardless of the presence of underlying comorbidities, and patients with SC disease were more likely to develop gram-negative bacterial and fungal infections. These findings may assist clinicians in the early identification of critically ill patients and the provision of appropriate empirical treatment.

## 1 Introduction

The global prevalence of influenza A virus (IAV) during winter is linked to high morbidity and mortality rates, with an estimated 3.2 million severe cases occurring annually. This poses a serious threat to global public health and economic stability worldwide (Luo et al., 2018; Ito et al., 2025). Despite the availability of effective vaccines and antiviral drugs, the World Health Organization estimates that between 290,000 and 650,000 people die of IAV infection annually. Owing to its pandemic potential, IAV was included in the World Health Organization’s priority pathogen list for 2024 (Jayadas et al., 2024).

Globally, the incidence of IAV infection exhibits a seasonal pattern, peaking in temperate regions during winter and coinciding with the monsoon season in tropical and subtropical regions (Jayadas et al., 2024). In China, the incidence of IAV infection was high from November 2024 to March 2025, prompting the National Health Commission to issue the 2025 Influenza Diagnosis and Treatment Plan. IAV primarily disrupts the respiratory tract through direct pathogenic effects, often leading to secondary bacterial infections, which, in turn, can lead to secondary viral infections. Most influenza-related deaths occur as a result of secondary bacterial pneumonia rather than as a direct effect of IAV infection (Shah et al., 2016; Shannon et al., 2022; Al-Dorzi et al., 2024; Santus et al., 2024; Valenzuela-Sanchez et al., 2024).

During the pandemic, the infection rate of H1N1 influenza A in the Community-Acquired Pneumonia region was 19%. Bacterial co-infection is more common in patients with H1N1 influenza pneumonia, with 18–33% of these patients experiencing bacterial co-infection. The most common isolated bacterial pathogens are *Streptococcus pneumoniae* (26.62%) and *Pseudomonas aeruginosa* (6.14%) (Estenssoro et al., 2010; Lee et al., 2010; Cilloniz et al., 2012). Since 2017 and especially after the COVID-19 pandemic, there has been a marked change in the epidemiological trends of influenza and other related respiratory pathogens, as well as changes in the clinical features of severe influenza (Wang et al., 2022; Hu et al., 2023). SARS-CoV-2 infection alters immune and metabolic responses in patients, which together produce an inflammatory environment that is highly permissive to fungal infections. The prevalence of COVID-19-associated mucormycosis in India was 0.27% in hospitalized patients. *Candida albicans* is the most frequently reported yeast species in critically ill patients with COVID-19 (44% of candidemia cases in one US multicenter study COVID-19-associated fungal infections) (Hoenigl et al., 2022; Seagle et al., 2022). There are currently no detailed reports on the co-occurrence of H1N1 influenza and fungal infection.

At present, the commonly used diagnostic methods for IAV pneumonia include the reverse transcription-polymerase chain reaction (PCR) test, which greatly improves the detection rate of IAV. However, there is still a partial false negative probability, which may be due to low early viral load. Additionally, mixed bacterial and fungal infections mask the characteristics of the virus, leading to the lack of relevant testing, which is also a reason for the delayed diagnosis of influenza A. At the same time, a single IAV reverse transcription-PCR test is powerless for the diagnosis of co-infections (Zhou et al., 2025). With the advancement of high-throughput sequencing technology, metagenomic next-generation sequencing (mNGS) has gradually progressed from initial use in microbiome research to wider clinical application. It played an important role during the COVID-19 pandemic, demonstrating the ability to quickly identify pathogens. Compared with traditional methods such as culture and PCR, mNGS has higher sensitivity, especially in immunocompromised individuals and individuals with complex co-infections (Graf et al., 2016; Shah et al., 2016; Huang et al., 2023). mNGS is useful for diagnosing pulmonary infections, especially mixed infections in immunocompromised patients (Zheng et al., 2025).

IAV infection can cause severe pneumonia and death, particularly in immunosuppressed individuals. Although studies suggest that viral and bacterial co-infections are common, the clinical importance of viral and bacterial co-infections remains poorly understood. Therefore, it is important to find an earlier and more effective method for diagnosing H1N1 pneumonia and secondary infections. This article retrospectively analyzed the data of our hospital’s diagnosis of influenza A virus pneumonia while excluding other virus co-infections, and clarified the practicality of mNGS in the diagnosis of influenza A virus pneumonia. Early identification of patients with severe and critical pneumonia, along with an analysis of the risk factors for secondary bacterial and fungal infections in patients with IAV pneumonia, is needed to provide a scientific basis for formulating effective prevention and treatment strategies and to reduce the length of hospital stay and mortality rates.

## 2 Methods

### 2.1 Study design and patients

This retrospective study included patients hospitalized at the Second Hospital of Jilin University between November 2024 and January 2025 and diagnosed with IAV pneumonia using the Influenza Diagnosis and Treatment Protocol (2025 version) (Influenza diagnosis and treatment plan, 2025). According to the protocol, patients were classified into two groups: MM and SC.

### 2.2 Eligibility criteria

All eligible patients hospitalized with IAV pneumonia were included in the analysis. Patients who had not undergone mNGS testing or had co-infections with other viruses were excluded. A total of 98 people completed the mNGS examination and confirmed that there was IAV infection. Among them, 25 people were excluded due to the presence of SARS-CoV-2 or other viruses, and a total of 73 people met the criteria for inclusion. On admission, all patients underwent chest imaging, including low-dose chest computed tomography or chest X-ray examination.

### 2.3 mNGS and influenza A testing method

On admission, mNGS was conducted on sputum or bronchoalveolar lavage fluid samples from all patients. We have previously reported the mNGS detection methods and reporting criteria (Yu et al., 2025).

### 2.4 Library preparation and metagenomic sequencing

A DNA library was prepared with automatic nucleic acid extraction, enzymatic fragmentation, end repair, terminal adenylation, and adaptor ligation according to a previous study (Luan et al., 2021). Finished libraries were quantified by real-time PCR (KAPA) and pooled. Shotgun sequencing was carried out on Illumina NextSeq. Approximately 20 million 50bp single-end reads were generated for each library. Bioinformatic analysis was conducted as described in a previous report (Shen et al., 2021). Briefly, sequences of human origin were filtered (GRCh38.p13), and the remaining reads were aligned to a reference database (NCBI nt, GenBank, and in-house curated genomic database) to identify the microbial species and read count. For each sequencing run, a negative control (culture medium containing 104 Jurkat cells/mL) was included.

### 2.5 mNGS reporting criteria

Microbial reads identified from a library were reported if: 1) the sequencing data passed quality control filters (library concentration > 50 pM, Q20 > 85%, Q30 > 80%); 2) negative control in the same sequencing run does not contain the species or the reads per million (sample)/reads per million (negative control) ≥ 5, which was determined empirically according to previous studies (Schlaberg et al., 2017; Wilson et al., 2019; Luan et al., 2021) as a cutoff for discriminating true-positives from background contaminations.

### 2.6 Influenza A antigen detection

To collect throat swabs for IAV testing, the swab tip was inserted into the throat, both tonsils and the mucosa of the posterior pharyngeal wall were wiped, and the swab was rotated 3 to 5 times and held for 5 to 10 s to adsorb the secretions. Colloidal gold-labeled immunochromatography was used to detect IAV.

### 2.7 Patient data collection

Data collected included sex, age, height, weight, comorbidities, and other basic information. Additionally, data were collected on laboratory test results, including routine blood test results, liver function test results, myocardial enzyme levels, procalcitonin level, C-reactive protein level, 1,3-β-D-glucan level, CT scans or radiographs, and arterial blood gas analysis results.

### 2.8 Treatment

All patients received oral oseltamivir along with symptomatic supportive treatment. Additionally, patients with hypoxemia or respiratory failure received supplemental oxygen via a nasal cannula, High-flow nasal cannula oxygen therapy, non-invasive ventilation, or invasive mechanical ventilation, as needed. Based on the Influenza Diagnosis and Treatment Protocol (2025 version), the treatment of co-infection is mainly determined through consultation between two doctors and clinical data. If it is a pathogenic bacterium, the corresponding treatment is given.

### 2.9 Ethics approval and informed consent

This study was approved by the Ethics Committee of the Second Hospital of Jilin University (Ethical approval number: 2024 166). All patients provided written informed consent for the publication of their personal and clinical details, including any identifying images. All methods adhered to the relevant ethical guidelines and regulations.

### 2.10 Statistical analysis

Statistical analyses were performed using SPSS (version 21.0, IBM Corp., Armonk, NY, USA). Normally distributed variables are presented as mean ± standard deviation, and groups were compared using t-tests. Categorical variables are presented as counts and percentages, and groups were compared using the Mann–Whitney U test. Partial Correlation Analysis was used to test the correlation. Results with *P* values < 0.05 were considered statistically significant.

## 3 Results

### 3.1 Patient characteristics, comorbidities, and pneumonia severity

**Figure 1:**
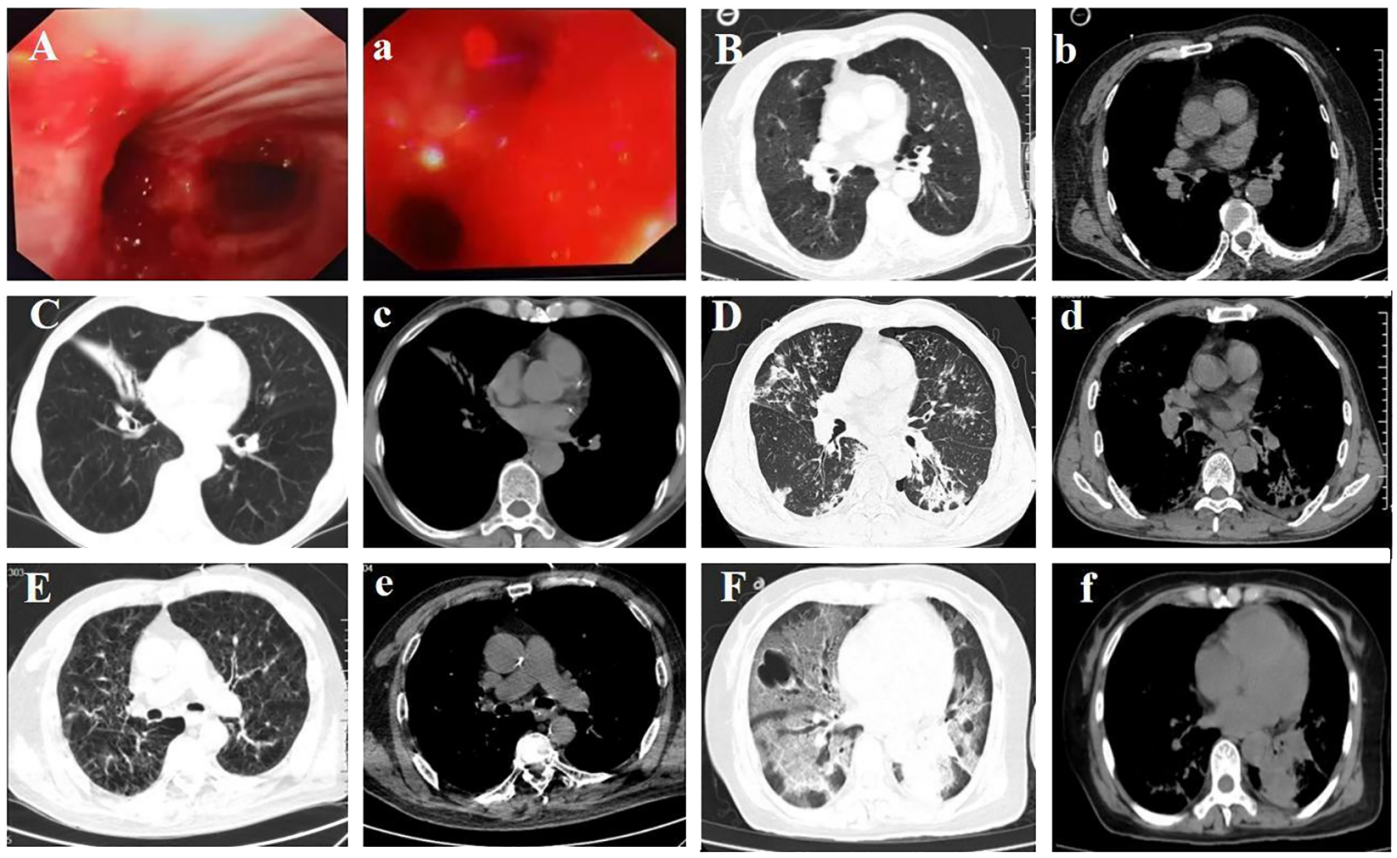
Diffuse alveolar hemorrhage and several typical lung CT findings of IAV pneumonia. **(Aa)**. Diffuse alveolar hemorrhage caused by IAV infection, as observed through fiberoptic bronchoscopy. **(Bb)**. IAV infection co-occurring with *Mycobacterium hominis* and *Streptococcus pneumoniae* infections. **(Cc)**. Patients with COPD and IAV infection complicated by *Pseudomonas aeruginosa* infection. **(Dd)**. Patients with COPD and IAV infection complicated by *Candida albicans* infection. **(Ee, Ff)**. IAV infection co-occurring with *Aspergillus* infection. CT, computed tomography; IAV, influenza A virus; COPD, chronic obstructive pulmonary disease, SP, *Streptococcus pneumoniae.*.

The study included 73 (42 male and 31 female) patients, with ages ranging from 29 to 88 years (mean: 62.3 years) (**Table 1**), of whom 45 (62%) were in the MM group and 28 (38%) were in the severe-to-critical (SC) group. Although the mean age of patients in the SC group was higher than that of patients in the MM group (65.5 versus 60.3 years), the difference was not statistically significant (*P* = 0.111). Almost all patients (72/73; 99%) presented with at least one respiratory symptom: cough, expectoration, fever, dyspnea, and hemoptysis, and one patient developed diffuse alveolar hemorrhage (**Figure 1A**). Consciousness disorders were also more common in the SC group than in the MM group (**Table 1**). The mean respiratory rate was significantly higher in the SC group than in the MM group (27.4 ± 4.4 versus 18.3 ± 2.7 breaths/min; *P* < 0.001; **Table 1**). Of the patients, 44 (60%) tested positive for IAV antigen on throat swabs. Laboratory investigations (**Table 2**) showed that neutrophil count, mean glutamic oxaloacetic transaminase level, and mean D-dimer level were higher in the SC group, but the difference was not statistically significant. The mean Procalcitonin level and lymphocyte count were lower in the SC group than in the MM group (*P* = 0.006 and *P* = 0.018, respectively). Compared with the MM group, the SC group had a significantly higher mean lactate dehydrogenase level (372 ± 199 U/L versus 235 ± 90 U/L; *P* < 0.001).

**Table 1:** Patient characteristics according to disease severity.

Variable	Total (N = 73)	Disease severity		*P* value
		Mild/ moderate (N = 45)	Severe/ critical (N = 28)	
Age (years), mean ± SD	62.3 ± 13.6	60.3 ± 13.8	65.5 ± 12.7	0.111
Age group				
<65 years	44 (60%)	30 (67%)	14 (50%)	0.157
≥65 years	29 (40%)	15 (33%)	14 (50%)	0.157
Sex				
Male	42 (58%)	24 (53%)	18 (64%)	0.361
Female	31 (42%)	21 (47%)	10 (36%)	0.361
BMI (kg/m^2^)	22.6 ± 5.3	22.8 ± 5.7	22.1 ± 4.3	0.591
Comorbidities	56 (77%)	31 (69%)	25 (89%)	0.046
Hypertension	25 (34%)	15 (33%)	10 (36%)	0.836
Cardiovascular disease	15 (21%)	9 (20%)	6 (21%)	0.884
Diabetes	17 (23%)	11 (24%)	6 (21%)	0.768
Malignancy	10 (14%)	5 (11%)	5 (18%)	0.418
Cerebrovascular disease	7 (10%)	3 (7%)	4 (14%)	0.286
Asthma or COPD	8 (11%)	1 (2%)	7 (25%)	0.003
Chronic kidney disease	1 (1%)	0 (0%)	1 (4%)	0.205
Bronchiectasis	1 (1%)	1 (2%)	0 (0%)	0.430
Rheumatic and immune system diseases	3 (4%)	3 (7%)	0 (0%)	0.166
Interstitial pneumonia	3 (4%)	1 (2%)	2 (7%)	0.306
Transplant	1 (1%)	0 (0%)	1 (4%)	0.205
Signs and symptoms	72 (99%)	44 (98%)	28 (100%)	0.430
Fever	49 (67%)	33 (73%)	26 (93%)	0.155
Cough	61 (84%)	37 (82%)	24 (86%)	0.697
Expectoration	56 (77%)	34 (76%)	22 (79%)	0.768
Dyspnea	37 (51%)	17 (38%)	20 (71%)	0.005
Disturbance of consciousness	5 (7%)	1 (2%)	4 (14%)	0.049
Respiratory rate (breaths/min)	21.8 ± 5.7	18.3 ± 2.7	27.4 ± 4.4	<0.001

Data are expressed as mean ± SD or d as n (%), n/N (%). Data were analyzed by t-tests or Mann-Whitney U test. BMI, body mass index; COPD, chronic obstructive pulmonary disease.

**Table 2:** Laboratory test results of 73 adult patients hospitalized with influenza A virus pneumonia, according to the disease severity.

Variable	Normal range	Total (N = 73)	Disease severity		*P* value
			Mild/Moderate (N = 45)	Severe/Critical (N = 28)	
Blood cells					
WBCs×10^9^/L	3.5–9.5	7.83 ± 4.33	7.48 ± 4.07	8.38 ± 4.73	0.390
Increased		21 (28.8%)	13 (28.9%)	8 (28.6%)	0.720
Decreased		10 (13.7%)	5 (11.1%)	5 (17.9%)	0.720
Neutrophils×10^9^/L	1.8–6.3	6.31 ± 4.46	5.58 ± 3.76	7.46 ± 5.26	0.081
LYs×10^9^/L	1.1–3.2	1.08 ± 0.69	1.31 ± 0.71	0.71 ± 0.48	<0.001
Increased		1 (1.4%)	1 (11.1%)2.2	0 (0%)	0.018
Decreased		45 (61.6%)	22 (48.9%)	23 (82.1%)	0.018
Neutrophil percentage	40%–75%	74.9 ± 14.9	69.8 ± 146	83.0 ± 11.7	<0.001
Lymphocyte percentage	20%–50%	17.2 ± 12.0	21.5 ± 12.6	10.6 ± 7.2	<0.001
Platelets×10^9^/L	125–350	221 ± 104	244 ± 113	185 ± 74	0.016
Hemoglobin, g/L	115–150	123 ± 24	122 ± 24	126 ± 24	0.539
Coagulation parameters					
Activated partial thromboplastin time, s	25.4–38.4	31.5 ± 3.2	31.6 ± 3.0	31.3 ± 3.6	0.639
Prothrombin time, s	9.4–12.5	11.8 ± 1.4	11.7 ± 1.3	11.9 ± 1.7	0.668
D-dimer, μg/mL	<0.5	2.1 ± 3.3	1.5 ± 3.2	3.0 ± 3.3	0.070
Blood biochemistry					
Glutamic-pyruvic transaminase U/L	7–40	34.6 ± 36.5	30.3 ± 21.6	40.9 ± 51.2	0.237
Glutamic oxaloacetic transaminase, U/L	13–35	33.2 ± 22.1	27.9 ± 17.5	41.3 ± 25.9	0.012
Lactate dehydrogenase, U/L	120–250	290 ± 157	235 ± 90	372 ± 199	<0.001
Infection-related biomarkers (increased)					
Procalcitonin, ng/mL	<0.5	20 (27.4%)	7 (15.6%)	13 (46.4%)	0.006
CRP, mg/L	<5	37 (50.7%)	19 (42.2%)	18 (64.3%)	0.069
1,3-β-D-glucan, pg/mL	<100.5	5 (6.8%)	2 (4.4%)	3 (10.7%)	0.306

Data are expressed as mean ± SD or d as n (%), n/N (%). Data were analyzed by t-tests or Mann-Whitney U test. CRP, C-reactive protein; WBC, white blood cells.

Of the patients, 56 patients (77%) had at least one comorbidity, with hypertension, diabetes, and cardiovascular disease being the most common comorbidities (**Table 1**). The proportion of patients with comorbidities was significantly higher in the SC group than in the MM group (25/28 [89%] versus 31/45 [69%]; *P* = 0.046; **Table 1**).

### 3.2 Co-infections identified in patients

**Figure 2:**
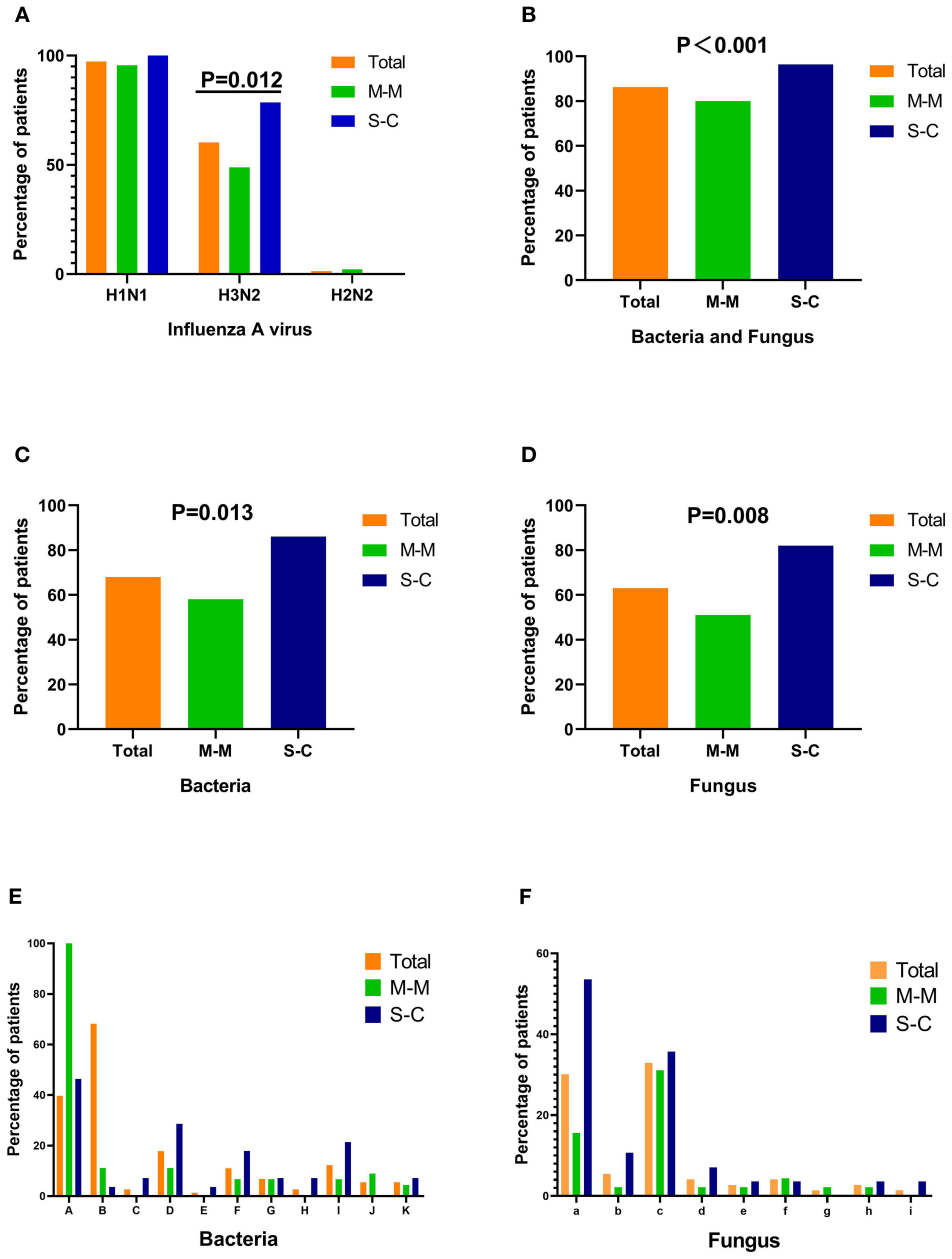
The percentage of patients infected with pathogenic bacteria and fungi. **(A)** The percentage of patients infected with influenza A virus subtypes. **(B)** The percentage of patients infected with bacteria or fungi in different groups. **(C, E)** The percentage of patients infected with different bacteria. **(D, F)** The percentage of patients infected with different fungi. M-M, mild/moderate group; S-C, severe/critical group; A, *Streptococcus pneumoniae*; B*, Staphylococcus aureus*; C, *Corynebacterium striatum;* D, *Klebsiella pneumoniae;* E, *Legionella pneumophila;* F, *Pseudomonas aeruginosa;* G, *Haemophilus influenzae*; H, *Stenotrophomonas maltophilia*; I, *Acinetobacter baumannii*; J, *Mycobacterium tuberculosis*; K, Nontuberculous mycobacteria*;* a, *Candida*; b, Yeast; c, *Aspergillus*; d, Thread fungi; e, *Rhizopus*; f, *Pneumocystis*; g, *Histoplasma capsulatum*; h, *Talaromyces marneffei*; i, *Fusarium oxysporum.*.

Most patients were infected with multiple IAV subtypes. The most identified subtypes using mNGS were H1N1 (71 cases, 97%) and H3N2 (44 cases, 60%). H2N2 was detected in only one patient (**Table 3**, **Figure 2**). H3N2 frequently co-occurred with H1N1 and was significantly more prevalent in the SC group than in the MM group (79% versus 49%, *P* = 0.012). Of the 73 patients, 63 (86%) had secondary or concurrent bacterial and/or fungal infections. Compared with the MM group, the SC group had a high prevalence of bacterial and fungal infections. The prevalence of gram-positive cocci was higher in the MM group than in the SC group, with *Streptococcus* being the most frequently detected. Conversely, the prevalence of gram-negative bacteria was significantly higher in the SC group than in the MM group (64% versus 24%, *P* = 0.001), with *Klebsiella pneumoniae* being the most frequently detected pathogen.

**Table 3:** Influenza A virus type and bacterial pathogens detected among 73 adult patients hospitalized with influenza A virus pneumonia, according to disease severity.

Pathogens identified	Total (N = 73)	Disease severity		*P* value
		Mild/ Moderate (N = 45)	Severe/ Critical (N = 28)	
Influenza A virus				
H1N1	71 (97%)	43 (96%)	28 (100%)	0.261
H3N2	44 (60%)	22 (49%)	22 (79%)	0.012
H2N2	1 (1%)	1 (2%)	0 (0%)	0.430
Bacteria and fungus	63 (86%)	36 (80%)	27 (96%)	<0.001
Bacteria	50 (68%)	26 (58%)	24 (86%)	0.013
Gram-positive coccus	34 (47%)	18 (40%)	16 (57%)	0.156
*Streptococcus pneumoniae*	29 (40%)	16 (36%)	13 (46%)	0.359
*Staphylococcus aureus*	6 (8%)	5 (11%)	1 (4%)	0.257
Gram-positive rods				
*Corynebacterium striatum*	2 (3%)	0 (0%)	2 (7%)	0.071
Gram-negative bacteria	29 (40%)	11 (24%)	18 (64%)	0.001
*Klebsiella pneumoniae*	13 (18%)	5 (11%)	8 (29%)	0.060
*Legionella pneumophila*	1 (1%)	0 (0%)	1 (4%)	0.205
*Pseudomonas aeruginosa*	8 (11%)	3 (7%)	5 (18%)	0.139
*Haemophilus influenzae*	5 (7%)	3 (7%)	2 (7%)	0.938
*Stenotrophomonas maltophilia*	2 (3%)	0 (0%)	2 (7%)	0.071
*Acinetobacter baumannii*	9 (12%)	3 (7%)	6 (21%)	0.064
*Mycobacterium*	8 (11%)	6 (13%)	4 (14%)	0.414
*M. tuberculosis*	4 (5%)	4 (9%)	0 (0%)	0.107
Nontuberculous mycobacteria	4 (5%)	2 (4%)	2 (7%)	0.625
Fungus	46 (63%)	23 (51%)	23 (82%)	0.008
*Candida*	22 (30%)	7 (16%)	15 (54%)	0.001
Yeast	4 (5%)	1 (2%)	3 (11%)	0.124
*Aspergillus*	24 (33%)	14 (31%)	10 (36%)	0.686
Thread fungi	3 (4%)	1 (2%)	2 (7%)	0.306
*Rhizopus*	2 (3%)	1 (2%)	1 (4%)	0.733
*Pneumocystis*	3 (4%)	2 (4%)	1 (4%)	0.856
*Histoplasma capsulatum*	1 (1%)	1 (2%)	0 (0%)	0.430
*Talaromyces marneffei*	2 (3%)	1 (2%)	1 (4%)	0.733
*Fusarium oxysporum*	1 (1%)	0 (0%)	1 (4%)	0.205

Data are indicated as n (%), n/N (%). Data were analyzed by Mann-Whitney U test.

The incidence of IAV infection with concurrent fungal infection was 63% (46 cases), making fungal infection the most common type of co-infection in both groups (MM group: 51%; SC group: 82%), regardless of the presence of comorbidities. *Aspergillus* was the most frequently detected fungal pathogen in both groups, whereas the prevalence of *Candida* infection was higher in the SC group than in the MM group (54% versus 16%; *P* = 0.001; **Table 3**, **Figure 2**).

### 3.3 Imaging data

Imaging revealed spot shadows, ground-glass opacities, and lung consolidation in cases of simple IAV infection. Suppurative lesions were observed in cases of bacterial co-infection (**Figures 1B** and **C**), whereas fungal infections presented with reversed halo signs and other characteristic features (**Figures 1D–F**).

### 3.4 Factors correlated with disease severity

Asthma and chronic obstructive pulmonary disease (COPD) were identified as independent risk factors for severe and critical IAV pneumonia (*P* = 0.004; **Table 4**). Neutrophil count and Procalcitonin, glutamic oxaloacetic transaminase, and lactate dehydrogenase levels were positively correlated with disease severity, whereas lymphocyte count was negatively correlated with disease severity. Both bacterial and fungal infections were positively correlated with disease severity (**Table 4**).

**Table 4:** Correlation of disease severity with different clinical parameters.

Variable	Correlation with disease severity	
	R	*P*
Respiratory rate	0.825	<0.001
Neutrophil percentage	0.438	<0.001
Lymphocyte percentage	−0.452	<0.001
Lymphocyte count	−0.423	<0.001
Platelet count	−0.283	0.016
Lactate dehydrogenase	0.402	0.001
Bacteria or fungi	0.244	0.038
Fungus	0.237	0.043
*Candida*	0.350	0.002

Data were analyzed using the partial correlation test.

### 3.5 Treatment-related outcomes

All patients received oral oseltamivir on admission. Those with prominent respiratory symptoms, such as fever, were administered antipyretic symptomatic treatment. Based on infection type, antibiotics (e.g., piperacillin, moxifloxacin, cefoperazone, and meropenem) and antifungal agents (e.g., fluconazole, voriconazole, and amphotericin B) were administered, along with other supportive treatments. Patients with hypoxemia or respiratory failure received respiratory support, including nasal cannula oxygen therapy, high-flow nasal cannula, non-invasive ventilation, and invasive mechanical ventilation. Length of stay was 12.59 ± 7.494 days. Four critically ill patients required mechanical ventilation and transfer to the intensive care unit (5.48%); the duration of mechanical ventilation was 20–336 h. Among these, one patient who discontinued treatment died, and the other two died despite receiving treatment (mortality rate: 4.11%). All the other patients survived.

## 4 Discussion

### 4.1 IAV clinical characteristics

In this study, H1N1 and H2N3 were identified as the most common IAV subtypes in our center for 2024-2025. However, as we are a single center study, the results may not represent the entire Jilin province. H2N3 often co-occurs with H1N1, and the incidence was higher in patients with critical disease severity. The incidence of severe and critical illness among hospitalized patients with IAV pneumonia was 38.4%, with some requiring mechanical ventilation. A strong association was observed between severe and critical illness and underlying conditions, particularly chronic airway diseases such as COPD and bronchial asthma. Common clinical symptoms observed included cough, expectoration, fever, and dyspnea. Hemoptysis also occurred in some patients, with one patient presenting with diffuse alveolar hemorrhage. Previous studies have linked IAV infection, especially H1N1 infection, to diffuse alveolar hemorrhage (Gilbert et al., 2010; Prasad et al., 2011; Toolsie et al., 2019), likely because of IAV-induced inflammatory storms in the lungs and immune dysregulation. The mortality rate among patients with diffuse alveolar hemorrhage secondary to IAV pneumonia is high (Toolsie et al., 2019). Treatment approaches include the use of glucocorticoids and immunosuppressants, and management of the primary infection (Park, 2013).

Many of the patients with severe and critical pneumonia presented with elevated neutrophil counts, which may be associated with co-infections. Additionally, the absolute lymphocyte counts were significantly reduced in patients with severe and critical pneumonia, which could serve as a predictor of disease severity. A lower lymphocyte count may indicate a more severe disease course, possibly owing to virus-induced lymphocyte apoptosis. The Fas-FasL signaling pathway plays a major role in lymphocyte apoptosis following IAV infection (Nichols et al., 2001). In this study, the D-dimer level was higher in patients with severe or critical pneumonia than in those with MM disease, suggesting a hypercoagulable state in patients with severe IAV infection. This is consistent with the previous reports by Kim et al. (Koh et al., 2012) and Assumpcao et al. (Talley and Assumpcao, 1971) that H1N1 infection can cause thrombotic diseases. Viral infections such as IAV and coronavirus (CoVs) can activate the coagulation system and lead to disseminated intravascular coagulation and thrombosis (Seagle et al., 2022). This reaction may be part of the host’s defense system to limit the spread of pathogens (Antoniak and Mackman, 2014). Multiple factors affect D-dimer, including cellular (endothelial cells and platelets) and protein (coagulation factors, anticoagulants, and fibrinolytic enzymes) components, which participate in the pathogenesis of influenza and COVID-19 during viral infection by enhancing viral replication and immune mechanisms. Endothelial dysfunction, severe hypoxia, and sepsis can all lead to coagulation dysfunction, and hypoxia-induced transcription factor-dependent signaling pathways can cause elevated D-dimer levels (Morris et al., 2021; Hu et al., 2023). Therefore, anticoagulant therapy should be considered during clinical treatment to prevent complications.

### 4.2 mNGS in IAV and co-infection

Currently, molecular detection of infectious diseases is largely pathogen-specific and requires the selection of the pathogen to be detected based on the clinical findings. Some infections, such as respiratory or urinary system infections, require multiple tests for diagnosis, and the sensitivity is limited. A previous study found non-targeted metagenomics detected 86% of known respiratory viral infections, whereas additional PCR tests only confirmed 33% of respiratory virus panel results in inconsistent samples (Graf et al., 2016). mNGS can be used for unbiased detection of any expected or unexpected pathogens. Compared with conventional microbiological tests alone, mNGS combined with conventional microbiological tests can enable earlier diagnosis and treatment, thereby reducing the time taken for clinical improvement in patients with severe pneumonia. mNGS is an important tool for detecting fungal, bacterial, and viral co-infections in patients with COVID-19 (Huang et al., 2023). The sensitivity of the metagenomic nanopore sequencing for detecting respiratory bacteria was 96.6%, whereas its specificity was only 41.7% (Charalampous et al., 2019). Our study included patients who were diagnosed with IAV based on mNGS and were confirmed to have influenza A pneumonia based on their clinical data. The IAV antigen positivity in these patients was only 60%. mNGS had greater sensitivity for IAV detection compared with IAV antigen testing. Additionally, mNGS provides information on IAV infection with bacterial co-infection. The sputum or Bronchoalveolar lavage fluid samples from hospitalized patients with IAV pneumonia were analyzed using mNGS in this study. The results showed that 86% of patients with IAV pneumonia had secondary or concurrent bacterial or fungal infections, a proportion substantially higher than that previously reported (Al-Dorzi et al., 2024). This may be because all patients in our study were hospitalized and had shown poor response to prior treatment. *S. pneumoniae* was the most common bacterial pathogen in this study. This finding differs from the results of previous studies, in which *Staphylococcus aureus* was more frequently observed as a bacterial co-infection (Bartley et al., 2022; Al-Dorzi et al., 2024). *Klebsiella pneumoniae*, *Acinetobacter baumannii*, *Pseudomonas aeruginosa*, *S. aureus*, and *Haemophilus influenzae* were other common secondary or concurrent bacteria in the patients. These findings differ from the findings of previous studies (Rice et al., 2012; Shah et al., 2016; Bartley et al., 2022; Al-Dorzi et al., 2024), possibly because of selection bias, as our institution is a referral hospital, and all patients in the study were inpatients. Additionally, in this study, patients in the MM and SC groups were infected with different bacterial species. Patients with MM disease severity were more commonly infected with gram-positive cocci, whereas those with severe or critical disease were more commonly infected with gram-negative bacteria. Compared with single *K. pneumoniae* infection, secondary infection following H9N2 IAV exposure exacerbated lung histopathological lesions and apoptosis, resulting in more severe disease. The mechanism is likely due to the virus promoting proliferation and delaying the clearance of *K. pneumoniae*. Secondary *K. pneumoniae* infection affects the transformation titer of serum anti-H9N2 antibodies and the cytokine profile (Li-Juan et al., 2022).

During the COVID-19 pandemic in 2021, combined fungal infections received increased attention. There are reports showing that the prevalence of COVID-19-related mucormycosis and *Candida albicans* infections in hospitalized patients is 0.27% and 44%, respectively (Hoenigl et al., 2022; Seagle et al., 2022). There is currently no clear statistical data on fungal infections related to H1N1 influenza. Our study also found that fungal infection was often secondary to or co-occurred with IAV infection and was not associated with the presence of comorbidities. *Aspergillus* was the most commonly identified fungal infection (33%), consistent with the findings of a previous study (Dou et al., 2022). IAV has been found to promote the growth of *Aspergillus fumigatus* through direct interaction *in vitro*. The incidence rate of influenza A pneumonia complicated with fungal infection was high, which may be because the cases were from a third-class hospital, and most of the patients admitted to the hospital were referred from other hospitals due to poor treatment outcomes. Therefore, the incidence rate of combined infection, especially fungal infection, is expected to be higher than previously reported. At the same time, it may also be related to the COVID-19 epidemic. Previous studies have illustrated the complexity of interactions between fungi and IAV (Shoji et al., 2021; Konig et al., 2024).

Gram-negative bacteria and fungi, particularly *Candida albicans*, were associated with severe and critical disease. This is consistent with previous research findings. Recent studies have found that IAV infections are frequently complicated by bacterial or fungal co-infections (Nickol et al., 2020). Secondary bacterial infection is recognized as a major complication of severe infection with H1N1 pdm09, contributing to high morbidity and mortality rates worldwide (Bal et al., 2020; Ishaqui et al., 2020). An interaction exists between influenza and certain bacteria, particularly *S. pneumoniae*, which exacerbates disease severity (Smith et al., 2013). IAV enhances *S. pneumoniae* colonization and infection (Diavatopoulos et al., 2010), resulting in the release of highly toxic pneumococci from biofilms *in vivo* and *in vitro* (Pettigrew et al., 2014). A mouse model study revealed that IAV infection facilitates *S. pneumoniae* colonization and proliferation by disrupting lung structure and suppressing humoral immunity. IAV infection may also enhance *S. pneumoniae* colonization and promote B lymphocyte suppression and depletion, leading to worsening pneumonia (Xiang et al., 2019). Viral–bacterial co-infections, such as IAV with *S. pneumoniae* infection, are well-documented causes of severe pneumonia (Rodriguez et al., 2019).

### 4.3 Limitation

This article is a single-center retrospective analysis. mNGS was conducted for all enrolled patients using sputum or Bronchoalveolar lavage fluid. We did not perform PCR or other nucleic acid tests for the H1N1 virus on the patients. Additionally, as our hospital is a tertiary hospital, most of the admitted patients received treatment from other hospitals, and their conditions continued to deteriorate. Therefore, there may be deviations in the clinical characteristics and laboratory tests, especially in cases of concomitant bacterial infections. mNGS has high sensitivity, but its low specificity cannot be ignored. Although we consulted with two or more physicians to confirm the pathogen, there may still be a possibility that some pathogens are colonizing bacteria. At the same time, we only collected patient data during the epidemic season, resulting in a small sample size; this may also cause some errors in the statistical analysis. Further improvements are needed in these areas in the future.

### 4.4 Conclusions

mNGS can enhance the sensitivity of influenza A diagnosis compared with IAV antigen testing and provide information on concurrent bacterial or fungal infections. H3N2 and H1N1 infections frequently occurred together in the 2024–2025 season, potentially contributing to critical pneumonia. Underlying diseases such as COPD and bronchial asthma, as well as increased respiratory rate, were associated with severe and critical IAV pneumonia. In clinical practice, patients with H1N1 pneumonia with these comorbidities are high-risk patients and need to be closely monitored for progression to severe pneumonia. *S. pneumoniae* was the most common co-infection microbiome, followed by fungi such as *Aspergillus* and *C. albicans*. IAV co-infection with fungi was common irrespective of the presence of comorbidities, these findings highlight the importance of considering fungal co-infections when selecting empirical treatment, particularly in severe or treatment-refractory cases. In addition, patients with severe or critical disease were more likely to develop infection with gram-negative bacteria, such as *K. pneumoniae* and *P. aeruginosa*. These combined infections suggest severe symptoms; these findings provide important guidance for empirical treatment in the future.

## Data Availability

The original contributions presented in the study are included in the article/supplementary material. Further inquiries can be directed to the corresponding author.
